# Millisecond-Timescale Local Network Coding in the Rat Primary Somatosensory Cortex

**DOI:** 10.1371/journal.pone.0021649

**Published:** 2011-06-29

**Authors:** Seif Eldawlatly, Karim G. Oweiss

**Affiliations:** 1 Department of Electrical and Computer Engineering, Michigan State University, East Lansing, Michigan, United States of America; 2 Neuroscience Program, Michigan State University, East Lansing, Michigan, United States of America; University of Michigan, United States of America

## Abstract

Correlation among neocortical neurons is thought to play an indispensable role in mediating sensory processing of external stimuli. The role of temporal precision in this correlation has been hypothesized to enhance information flow along sensory pathways. Its role in mediating the integration of information at the output of these pathways, however, remains poorly understood. Here, we examined spike timing correlation between simultaneously recorded layer V neurons within and across columns of the primary somatosensory cortex of anesthetized rats during unilateral whisker stimulation. We used Bayesian statistics and information theory to quantify the causal influence between the recorded cells with millisecond precision. For each stimulated whisker, we inferred stable, whisker-specific, dynamic Bayesian networks over many repeated trials, with network similarity of 83.3±6% within whisker, compared to only 50.3±18% across whiskers. These networks further provided information about whisker identity that was approximately 6 times higher than what was provided by the latency to first spike and 13 times higher than what was provided by the spike count of individual neurons examined separately. Furthermore, prediction of individual neurons' precise firing conditioned on knowledge of putative pre-synaptic cell firing was 3 times higher than predictions conditioned on stimulus onset alone. Taken together, these results suggest the presence of a temporally precise network coding mechanism that integrates information across neighboring columns within layer V about vibrissa position and whisking kinetics to mediate whisker movement by motor areas innervated by layer V.

## Introduction

The massive size of neocortical networks, their convergent-divergent links and their nested feedback loops suggest the vastly complex information processing mechanism that underlies their operation. In the rat primary somatosensory (barrel field) cortex (S1), neurons are known to encode vibrissa movements in their individual firing patterns [1]–[3]. It has been suggested that precise spike timing relative to stimulus onset carries most of the information about whisker displacement [4], and that this mechanism aids the animal during active whisking episodes to recognize external objects. This mechanism is primarily present in layer IV as this layer receives numerous inputs from the ventral posterial medial (VPM) thalamus through the lemniscal and paralemniscal pathways [3], [5]. Neurons in infragranular layer V, on the other hand, exhibit more complex dynamics as they integrate inputs from multiple barrels within and across hemispheres [6]–[8]. This integration creates larger receptive fields than those typically found in layer IV cells [9], [10].

Because layer V is a major output layer to multiple structures such as the posterior medial nucleus, zona incerta, pontine nuclei and the primary motor cortex [11]–[13], the frequently observed stimulus-dependent correlation among layer V cells is believed to play an important role in providing two streams of information [14]: a spatial code from ascending pathways representing current whisker position, and a corticothalamic feedback representing information about past whisker position [15]. These two streams are necessary to provide sufficient information to primary motor cortex for mediating active whisking cycles during adaptive exploratory behavior of objects, much like the dexterous control of hand digits by primates [16].

Despite the large body of reports suggesting that individual layer V neurons encode spatial information, the precise mapping of this spatial information to temporal coordination among these neurons during whisker movements remains poorly understood. In this study, we examined how the precise spike-by-spike correlation among multiple, locally observed layer V cells plays a role in encoding whisker movement. We simultaneously recorded layer V multiple single unit activity in anesthetized rats during unilateral mechanical stimulation of individual whiskers. We analyzed the firing patterns of these units by fitting dynamic Bayesian network (DBN) models to the spike trains sampled at sub-millisecond time scale [17]–[20]. Model fit represented the effective connectivity between the cells and resulted in networks expressing whisker-specific, causal influence between their individual outputs. We hypothesized that for a given whisker movement, a stable network representation would be obtained - as measured by the degree of structural similarity between individual networks inferred across multiple repeated trials. In addition, the structure of these networks would be less similar to those inferred during mechanical stimulation of other whiskers.

Our results demonstrate that stable, whisker-specific, local network structures were present. Moreover, the categorization of putative pre- and post-synaptic cells in these networks was strongly consistent with the cells' post stimulus first spike latency. Specifically, cells with shorter response latency were mostly pre-synaptic, while those with longer response latency were mostly post-synaptic. Furthermore, the inferred networks could be efficiently used to decode the identity of the deflected whisker with much higher accuracy compared to the case when the first-spike latency was the only feature used. Finally, prediction of the temporally precise firing pattern of putative post-synaptic cells using pre-synaptic cell activity was achieved with much higher fidelity than using stimulus onset time only. These findings suggest that network population coding in the somatosensory cortex occurs at a much finer temporal and spatial resolution than previously thought, and that this highly coordinated encoding mechanism relies on relatively few - but rather strong - connections between population elements. These results are consistent with previous reports demonstrating similar mechanisms in other cortical layers and across many species [21]–[26], albeit at a much coarser temporal resolution, suggesting that localized network codes are a universal encoding mechanism for mediating information flow across many neocortical structures.

## Results

### Firing Characteristics

**Figure 1:**
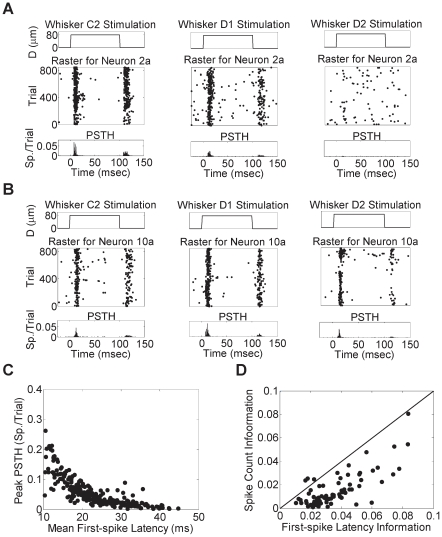
Firing characteristics of the recorded neurons. Two sample neurons: (A) Neuron 2a and (B) Neuron 10a from rat R2 response to stimulation of whiskers C2, D1 and D2. (Top) Whisker displacement. (Middle) Spike raster over multiple repeated trials. (Bottom) Post-stimulus Time Histogram (PSTH) with 0.5ms bin size. Neuron 2a shows stronger and faster response to whisker C2 than other whiskers while Neuron 10a shows a slightly stronger and faster response to whisker D1. (C) Peak PSTH counts of each neuron for each whisker versus its mean first-spike latency (*n* = 240). Each dot corresponds to the response of a single neuron to a single whisker. (D) Normalized mutual information between spike count/first-spike latency and whisker identity. Each dot corresponds to one neuron. Black diagonal line represents equal mutual information (*n* = 80).

We recorded a total of 80 single units from layer V of the barrel cortex in five anesthetized rats (R1–R5). Microelectrode arrays with 32 channels were used in each rat to record neural responses to unilateral stimulation of individual whiskers, one at a time. Each whisker was stimulated 900 times at a frequency of 1 Hz. Compared to previous studies that addressed individual neurons' firing characteristics in which only 50 trials were used [1], [4], [9], we used this large number of trials (900 trials) to guarantee a sufficiently large sample size to infer causal networks. Figure 1 illustrates the discharge patterns of two sample neurons during the deflection of three whiskers in rat R2. Onset response latencies were in the range of 10+ ms, corresponding to typical response patterns of pyramidal cells in layer V of the barrel cortex [27], [28]. Neurons showed significant preference to modulate their firing pattern in response to whisker-specific deflection, with 71.3% of the recorded units exhibiting a significantly stronger response to stimulation of a principal whisker during the first 100 ms post stimulus onset compared to other non-principal whiskers (*P*<0.05, two-sample *t*-test for each pair of whiskers).

It has been reported that the first spike post-stimulus onset in a barrel column conveys most of the information about the corresponding whisker deflection [4], [29]. Therefore, we used the post stimulus first-spike latency as a measure of tuning as well as temporal response fidelity of the recorded neurons. We found that 83.8% of the recorded neurons had a significantly smaller first-spike latency for a single whisker compared to other whiskers (*P*<0.05, two-sample *t*-test for each pair of whiskers). A fraction of the recorded units (46.3%) showed preference to the same whisker in terms of both firing rate and first-spike latency, suggesting the presence of both rate and temporal coding mechanisms [30]. Neurons with strong response modulation to a given whisker also exhibited short latency as illustrated in Figure 1C (*r* = −0.76, *P*<0.0001, *n* = 240, *t*-test).

To confirm the salience of spike timing in encoding whisker movements, we computed the mutual information between the stimulus and each individual response property (namely, the first-spike latency and the spike count) [4], [31]. More information was conveyed about the stimulus by the first-spike latency than the spike count as illustrated in Figure 1D (*P*<0.0001, *n* = 80, two-sample *t*-test). Indeed, 96.25% of the recorded neurons had larger first-spike latency information than spike count information, indicating that temporal coding was more pronounced compared to rate coding (Information in first-spike latency: 0.19±0.08 bits, information in spike count: 0.05±0.04 bits, Normalized information in first-spike latency: 0.04±0.02, normalized information in spike count: 0.01±0.01, mean ± SD).

### Whisker-specific Networks

As illustrated in Figure 1, variability in the temporal characteristics of the responses across whiskers was observed. We asked whether this variability could be accounted for using a network model of the recorded ensemble beyond what is provided by individual neurons' response variability. To address this question, we analyzed the data by fitting a dynamic Bayesian network (DBN) model [17]. Unlike pair-wise metrics of connectivity such as cross-correlograms or directed coherence [20], a unique advantage of DBN is its ability to *explain away* unlikely causes of firing while taking into account the activity of the entire observed population. This enables DBN to identify direct - and possibly nonlinear - coupling between neurons and rule out possible indirect, or spurious, connections that may be inferred, for example, due the presence of a common observed input to the cells.

**Figure 2:**
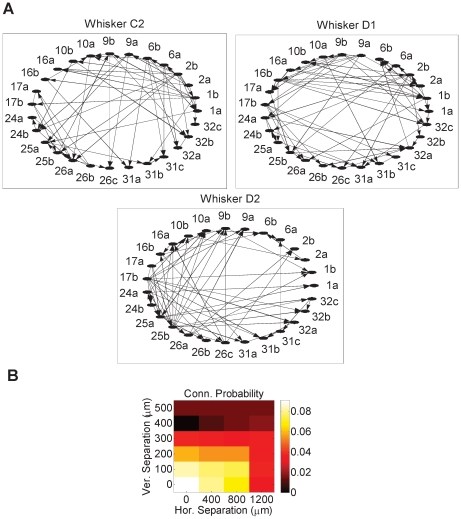
Sample whisker-specific networks. (A) DBN networks inferred from one population (Rat R2) for 3 individually stimulated whiskers: C2, D1 and D2. Undirected links indicate bidirectional connections. Network of each whisker was inferred from a dataset of length 18 sec (180 trials x 100 ms). (B) Connection probability in the DBNs as a function of the horizontal and vertical separations between the electrodes on which neurons were recorded. The number of connections inferred at each distance was normalized by the corresponding total number of possible connections.

For each stimulated whisker, one hundred 18-sec long spike train datasets were formed from the 900 trials of each whisker by randomly sampling 180 trials out of the 900 trials following a uniform distribution. DBN fit was then obtained for each of these datasets. Figure 2A illustrates sample inferred networks for three distinct whiskers in one rat. To assess the validity of these networks in the absence of knowledge of the underlying true connectivity, we examined the connection probability as a function of the horizontal and vertical separation between electrodes. We expected that neurons recorded on the same or adjacent electrodes are more likely to be connected in the inferred networks, consistent with anatomical and physiological studies in the neocortex suggesting that connectivity tends to be mostly local for economic wiring [32]–[34]. Figure **2**B demonstrates that neurons recorded on the same or on adjacent electrodes have a higher probability of being connected. Furthermore, the connection probability decreased with increasing electrode separation (*r* = −0.47, *P*<0.05, *n* = 24, *t*-test).

**Figure 3:**
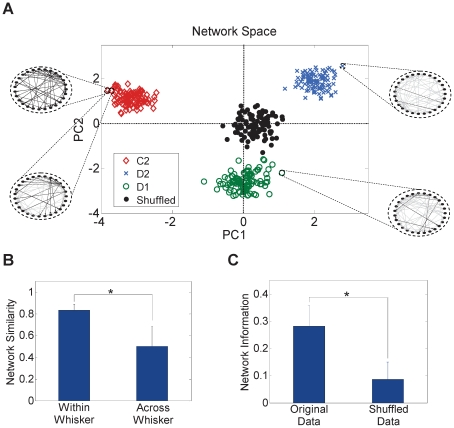
Networks similarity within- and across-whiskers. (A) Network feature space of rat R2 for 3 different whiskers (C2, D2 and D1) and a shuffled dataset. Each dot corresponds to the projection of one network onto a 2-dimension principal components (PC1 and PC2) feature space. Insets: Sample networks from the 3 whiskers. Black edges represent common connections with the top left sample network (whisker C2). (B) Similarity between networks inferred for the same whisker and between networks inferred for different whiskers averaged across subjects (mean ± SD). Similarity for a given pair of networks was quantified as 1 – the normalized distance between the projections of the pair in the principal component space. Within whisker similarity was corrected for the bias resulting from the overlap in the data (estimated from the shuffled data). The figure indicates that the within-whisker networks cluster more closely compared to across-whisker networks. *P<1*e*-6, two-sample *t*-test. (C) Normalized mutual information between each of the networks inferred from the original and shuffled data, and the stimulus averaged across subjects (mean ± SD). *P<0.001, two-sample *t*-test.

To examine whether the inferred networks are indeed whisker-specific, we compared the similarity between the networks inferred for the same whisker (termed herein *within-whisker* similarity) to the similarity between the networks inferred for different whiskers (termed *across-whisker* similarity). To do this, principal component analysis (PCA) was used to construct a feature space of these networks, where each point in that space corresponded to one network as illustrated in Figure 3A [35]. We quantified the similarity between a pair of networks as (1 – the normalized distance between their corresponding projections in the PCA network space). Distance normalization ensured that the maximum possible pair-wise distance measure in the network feature space did not exceed ‘1’. As illustrated by Figure 3B, networks inferred for the same whisker were significantly more similar (closer in the feature space) compared to those inferred for other whiskers (83.3±6% within whisker, 50.3±18% across whiskers, *P*<1*e*-6, two-sample *t*-test). This suggests that the inferred networks are whisker-specific, and that temporal coordination among the observed neurons bore a signature of the deflected whisker identity.

We next examined whether the overlap between the datasets extracted for the same whisker (20±4% overlap between any given pair of datasets) may have resulted in a statistical bias towards the within-whisker similarity measures. We estimated that bias by creating multiple surrogate datasets for each population. Each surrogate dataset consisted of 900 trials, 300 trials from each whisker. One hundred 18-sec long datasets were then extracted from each surrogate dataset after shuffling in the same way as the original 100 datasets/whisker were extracted. This shuffling procedure destroyed whisker-specific features within each dataset but maintained the same amount of overlap (Figure S3). Therefore, any similarity within each surrogate dataset that exceeds similarity across surrogate datasets would only result from the overlap between the datasets within each surrogate. We found the bias of within-whisker similarity to be 1.8±3% (Figure S3). In addition, the projection of the networks inferred from the surrogate data clustered around the origin in the network space as seen in Figure 3A. This demonstrates that the networks inferred from the shuffled data represented pure noise and confirmed that the networks inferred from the original data were whisker-specific (distance from the origin for the original data: 1.83±0.9, distance from the origin for the surrogate data: 0.75±0.5, *P* = , two-sample *t*-test).

We then examined the amount of information conveyed by the networks about the stimulus and compared it to those conveyed by single individual neuron responses. Averaged across subjects, information of 0.28±0.08 (Un-normalized: 1.4±0.3 bits) was obtained from the original data compared to only 0.09±0.07 (Un-normalized: 0.28±0.28 bits) from the shuffled data. The latter could also be used as an estimate of the bias resulting from the overlap between within-whisker datasets (Figure 3C). Therefore, after correcting for the bias, the inferred whisker-specific networks convey an average net normalized information of ∼0.2. Compared to individual neurons' response information, we found network information to be approximately 6 times the information provided by the first-spike latency and 13 times the information provided by the spike count. This suggests that the network code provides orders of magnitude more information about the stimulus compared to rate and temporal codes combined.

**Figure 4:**
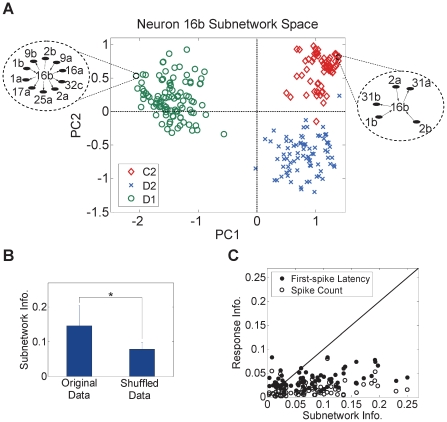
Individual neuron subnetwork information. (A) Network feature space of one sample neuron (Neuron 16b of rat R2) for 3 different whiskers (C2, D1 and D2). Each dot corresponds to the projection of one network onto a 2-dimension principal components (PC1 and PC2) feature space. (B) Normalized information in the individual neurons' networks inferred for the shuffled data versus the original data (mean ± SD). * *P* = 0, two-sample *t*-test. (C) Normalized information in first-spike latency and spike count versus information in the networks of each neuron. Each dot corresponds to one neuron. Black diagonal line represents equal information. Network information was corrected for any statistical bias by subtracting the network information computed for each neuron from the shuffled data in (B).

Within each of the networks inferred, some neurons exhibited strong participation in subnetworks. Here we sought to infer how much information about the stimulus was conveyed in each neuron subnetwork compared to its individual response characteristics. This was done by, first, extracting the subnetwork where each neuron was an actual element, and second, computing the principal components (PCs) of these subnetworks as illustrated in the example of Figure 4A. Individual neurons' subnetworks conveyed more information about the stimulus than individual responses (first-spike latency and spike count), albeit at a reduced precision compared to the entire network case as illustrated in Figure 4B (Normalized information for original data: 0.15±0.06, shuffled data: 0.08±0.02, *P* = 0, two-sample *t*-test; Un-normalized information for original data: 0.82±0.3 bits, shuffled data: 0.4±0.2 bits, *P* = 0, two-sample *t*-test). Moreover, 78.6% and 94.3% of the neurons conveyed more information through their subnetworks than their individual first-spike latency and spike count, respectively, as can be seen in Figure 4C. This suggests that the network code provides better stimulus discrimination than rate and temporal codes at the local subnetwork level as well as the global population level.

The significant amount of information conveyed by the network suggests that decoding whisker identity based on network features would be more accurate. For each test dataset, the identity of the deflected whisker was decoded as the whisker whose inferred network had the highest similarity to the network inferred from the training dataset (using a leave-one-out cross-validation method). Overall, decoding accuracy reached 97.6±3% across subjects (1464 of 1500 datasets were classified correctly), compared to only 79.7±12% when the response latency of each neuron was used as a feature for decoding, whereas using a *majority voting* method using all neurons reached an accuracy of 87.7±13%.

### Relating Network Properties to Individual Neuronal Responses

**Figure 5:**
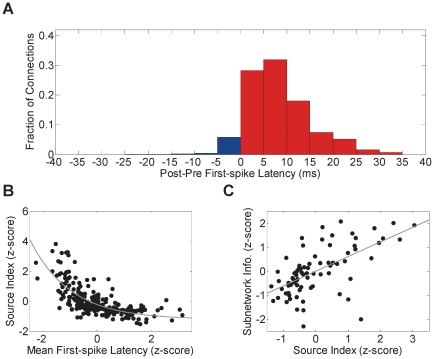
Network properties and individual neuronal responses. (A) Histogram of the difference between the mean first-spike latency of the post-synaptic cells and the pre-synaptic cells for each inferred connection. Only unidirectional connections were counted in the histogram. Red bars indicate the fraction of connections consistent with the difference between latencies while blue bars indicate connections that are not. (B) Ratio between the number of outgoing connections and incoming connections for each neuron (source index) as a function of its mean first-spike latency for each whisker. Each dot corresponds to one neuron for a given whisker (*n* = 240). Z-scores of the mean first-spike latency and the source index are reported on the X-axis and the Y-axis, respectively. Gray curve indicates decaying exponential fit. (C) Information in the networks of each neuron as a function of the source index averaged across whiskers (*n* = 80). Each dot corresponds to the standardized z-scores of one neuron. Gray line indicates regression line.

From the causal DBN fit, neurons could be categorized as putative pre- and post-synaptic cells. To examine whether this categorization is consistent with individual neuron response properties, we first examined whether the direction of an inferred connection between a given pair of neurons is consistent with the sign of the difference between their mean first-spike latencies. As Figure 5A illustrates, we found that for 93.3% of the inferred unidirectional connections, neurons categorized as pre-synaptic cells had smaller latencies than neurons categorized as post-synaptic cells.

Neurons could also be categorized as sources and sinks, based on the number of outgoing (fan-out) and incoming (fan-in) connections in the DBN fit, respectively. We tested this categorization by finding the relationship between the ratio of the number of outgoing to incoming connections for each neuron, termed *the source index*, and the response latency. Figure 5B illustrates the source index as a function of the first-spike latency. This index decays exponentially with the mean first-spike latency (Time constant  = −0.67, *r* ^2^ = 0.53, *n* = 240, *t*-test). Neurons with relatively large number of outgoing connections constituted central hubs in the network, thereby acting as “source” nodes (as can be seen in Figure 2A). Neurons with high source index were found to convey more information about the stimulus than neurons with low source index as seen in Figure 5C (*r* = .61, *P*<1e-8, *n* = 80, *t*-test). The identity of these source neurons was again whisker–specific (for e.g., neuron 2b for whisker C2, neuron 16b for whisker D1, and neuron 17b for whisker D2) and was highly correlated with short response latency. On the other hand, neurons with large response latency were observed to have more incoming connections, thereby acting as “sink” nodes. One way to interpret these observations is that neurons with short response latency receive information about whisker deflection *before* neurons with larger response latency. This suggests that few, strongly connected hub neurons are key players in orchestrating the local population response to the stimulus.

### Cross-correlogram Comparison

**Figure 6:**
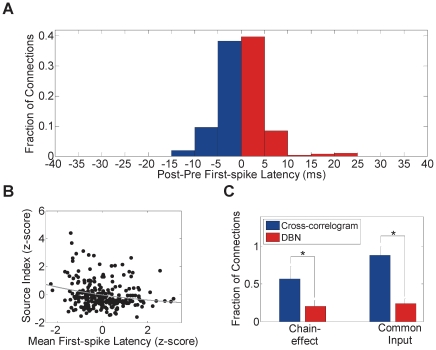
Comparison with networks inferred using cross-correlograms. (A) Histogram of the difference between the mean first-spike latency of the post-synaptic neurons and the pre-synaptic neurons for each connection inferred using the cross-correlogram technique. Only unidirectional connections were counted in the histogram. Red bars indicate the fraction of connections consistent with the difference between latencies while blue bars indicate connections that are not. (B) Ratio between the number of outgoing connections and incoming connections for each neuron (source index) as a function of its mean first-spike latency for each whisker. Each dot corresponds to one neuron for a given whisker (*n* = 240). Z-scores of the mean first-spike latency and the source index are reported on the X-axis and the Y-axis, respectively. Gray curve indicates decaying exponential fit. (C) Fraction of possible chain effect-induced connections and common input-induced connections inferred by cross-correlogram and DBN (mean ± SD). * *P*<0.001, two-sample *t*-test.

The cross-correlogram is a classical method to identify functional connectivity between cells over very short time scales (<5 ms) [32], [36]. Cross-correlogram analysis of our data revealed some similarity - but also some substantial differences - compared to the DBN analysis. In particular, Figure 6A shows the connection probability calculated from cross-correlogram analysis as a function of the difference between post and pre-synaptic neuron latencies. Contrary to the DBN result in Figure 5A, the probability of inferring a connection from small latency neurons to large latency neurons was not significantly higher than inferring a connection in the opposite direction (Cross-correlogram: 50.1%, DBN: 93.3%). Similar to Figure 5B, Figure 6B shows a negative correlation between the source index and response latency but less significant than what was obtained using DBN analysis (Cross-correlogram: time constant  = −0.23, *r* ^2^ = 0.04, *n* = 240; DBN: time constant  = −0.67, *r* ^2^ = 0.53, *n* = 240). The cross-correlogram analysis, however, revealed some inconsistency with individual neuron response analysis. In particular, more connections were observed to be inconsistent with the response latency of individual neurons, suggesting they represent spurious connections. From a statistical standpoint, this was not surprising because, in contrast to DBN, the cross-correlogram method – by virtue of the fact that it is a pair-wise measure – does not *explain away* unlikely causes of correlation. To demonstrate that this is indeed the case, consider a simple example of 3 neurons A, B and C forming a chain, where A→B and B→C but no connection exists between A and C. A pair-wise measure such as the cross-correlogram would infer the direct connection A→C. In the case of the common input A driving both B and C, the cross-correlogram would detect the spurious connections B→C or C→B. We quantified the ability of the DBN method to infer direct causal influence in the 3-neuron chain case as well as the common input case and compared it to the cross-correlogram method. As shown in Figure 6C, we found that the cross-correlogram inferred a significantly higher number of spurious connections compared to DBN (*P*<0.001, two-sample *t*-test). On average, the cross-correlogram inferred 36.2% more connections than DBN for the 3-neuron chain case, and 64.4% more connections for the common input case. These results suggest the limited ability of the cross-correlogram in inferring effective connectivity between simultaneously observed neurons.

### Network Model-based Prediction of Single Neuron Firing

**Figure 7:**
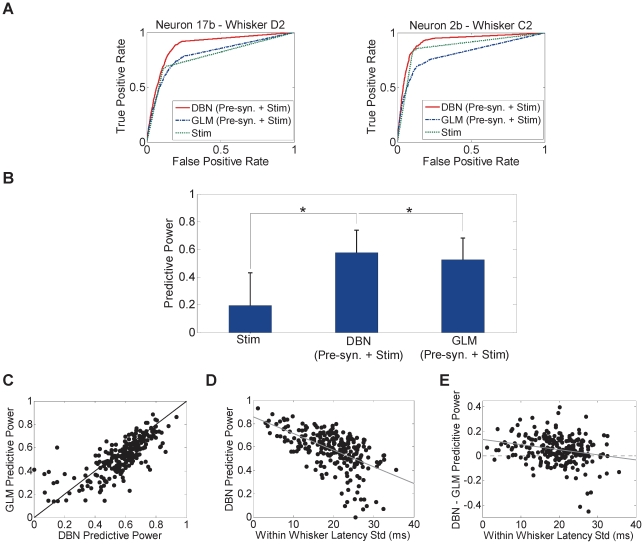
Predicting single neuron firing. (A) ROC curves of sample neuron 2b in response to whisker C2 deflection (left) and neuron 17b in response to whisker D2 deflection (right) in rat R2. Predicted spike trains were obtained using the pre-synaptic cells' history inferred by DBN and stimulus onset time, pre-synaptic cells' history inferred by the GLM and stimulus onset time, and the stimulus onset time only. Each curve was computed from tenfold cross-validation datasets. (B) Predictive power comparison across all rats for multiple models (mean ± SD). * *P*<0.001, two-sample *t*-test. (C) GLM predictive power versus that obtained using DBN and stimulus onset time. Black diagonal line represents equal prediction (*n* = ). (D) Predictive power obtained using DBN and stimulus as a function of the variability in mean first-spike latency. (E) Difference between predictive power for each neuron obtained using DBN and stimulus and that obtained using GLM as a function of the variability in mean first-spike latency. Each dot corresponds to one neuron for a given whisker (*n* = 240). Gray lines in (D) and (E) indicate regression lines.

Prediction of neuron firing is a well-established method to measure a model's goodness of fit. We therefore examined the ability of the inferred networks to predict the firing of individual neurons. The probability of firing of each neuron at any given time point was estimated based on the firing history of the neuron's pre-synaptic connections as determined by the network structure for a given whisker dataset and stimulus onset. Predicted spike trains were obtained by comparing the probability of firing to a variable threshold (see Materials and Methods) and computing the percentage of times the spikes in the predicted spike train matched the original spike train (True positives) and the percentage of times they did not match (False positives) for each threshold value [37]. We then compared the Receiver Operating Characteristics (ROC) obtained using DBN fit to those obtained using another known statistical model – the Generalized Linear Model (GLM) [38] - conditioned on pre-synaptic cell history and stimulus. GLMs have been shown to fit spike train data in a number of brain structures [37]–[40]. Figure 7A demonstrates sample ROC curves of two cells. As can be seen, the predictive power of DBN conditioned on the pre-synaptic cells' history as well as the stimulus onset was the highest, suggesting that network model fit using DBN was more accurate.

To quantify the predictive power for each neuron, we used the area under the ROC curve (AUC) [37]. An AUC of 0 indicates a prediction that is similar to chance level, while an AUC of 1 indicates perfect prediction, with a highly deterministic conditioned response. Figure 7B shows a comparison between the predictive power of different models. DBN predictive power conditioned on the history of pre-synaptic cell firing and the stimulus onset time was significantly higher than that obtained conditioned on the stimulus onset time only (stimulus only: 0.2±0.24, DBN pre-synaptic cells' history and stimulus: 0.58±0.16, *P* = 0, two-sample *t*-test). The predictive power of the DBN was slightly higher than that of the GLM (GLM: 0.53±0.16, *P*<0.001, *n* = 240, two-sample *t*-test) as illustrated in Figure 7C. DBN predictive power was inversely proportional to the variability in response latency as seen in Figure 7D (*r* = −0.56, *P*<1*e*-20, *n* = 240, *t*-test). Thus, better overall prediction was obtained for cells with small variance in the response temporal precision compared to cells with high variance. For this group of cells, DBN prediction was better than GLM as shown in Figure 7E (*r* = −0.26, *P*<0.001, *n* = 240, *t*-test).

## Discussion

Neural coding theories in the sensory neocortex posit that fast integration of sensory information is crucial to the organism's ability to guide motor actions. In the rat somatosensory cortex, previous studies have consistently demonstrated that putative pyramidal neurons in layer IV receiving input from trigeminal nuclei through VPM thalamus showed the prevalence of temporal coding over rate and correlation coding [4], [9], [30], [41], [42]. Whether temporal coding at the population level provides sufficient information to subserve the rapid sensorimotor integration mechanisms needed to perform active whisking remained poorly understood.

Here, we examined the dynamics of spike timing correlation between local, simultaneously observed neurons in layer V in response to unilateral whisker stimulation. We showed that rapid network dynamics between these neurons, as determined by the stable, whisker-specific dynamic Bayesian networks, provided evidence of a synergistic code that mediates information flow. Furthermore, we were able to demonstrate that the effective connectivity revealed by the structure of these networks provided more information about the stimulus than what was provided by both temporal and rate codes of each neuron analyzed individually. In particular, we showed that the decoding performance of these networks was ∼18% higher compared to that of the first-spike latency, and that the inferred connections were important in predicting individual neurons' firing patterns. This agrees with previous studies that addressed similar questions in other brain areas [39], [43], [44], albeit at a much coarser temporal and spatial resolution.

It is widely accepted that complex, possibly nonlinear, response characteristics occurring at the population level such as those described here are more pronounced in higher cortical areas such as the prefrontal cortex, or subcortical areas such as the hippocampus and the thalamus [32], [45], [46]. These characteristics have been also hypothesized to underlie the fast oscillatory patterns observed throughout many cortical layers [47], [48]. These patterns are known to originate in the cortex and do not require rhythmic drives from the thalamus, as demonstrated by direct electrical stimulation studies [16], [49]. Here we demonstrated that the origin of these dynamics may be rooted in the precise temporal coordination among layer V neurons, possibly to subserve the integration of multiple information processing streams needed to mediate sensorimotor transformations by areas innervated by layer V, and in particular the motor cortex. Whether confirming that the connectivity inferred in this study represents actual anatomical connectivity between the cells was not possible with our recording or analysis methods. Nevertheless, there is substantial evidence in the literature indicating that local anatomical connectivity in sensory cortices is predominantly present [50], [51], and our results provide strong support of these findings.

A plausible interpretation of our findings would be that since layer V is a major output of the barrel cortex to other brain areas such as the thalamus, the motor cortex and the pontine nuclei [11], information has to be rapidly integrated across cortical columns to regulate the whisking behavior needed to discriminate between different objects that come in contact with the whiskers [15]. Thus, these high-level functions might require a *gain-modulation* mechanism that is dynamically shaped by a variable number of participating neurons coordinating their information-bearing signals to represent stimulus attributes that cannot be provided by single units responding individually to their principal whiskers. Our results therefore suggest a strong account for a synergistic coding mechanism in layer V that may reflect stimulus-dependent states of the observed population. While these findings agree with previous reports of multi-whisker integration in layer V of the barrel cortex at the single cell level [9], [11], it provides the first evidence that this integration occurs at the network level within millisecond timescales.

It should be noted that modulation of the firing rates of S1 neurons in response to whisker deflection is known to be relatively weaker in the sleep state (here under anesthesia) than in the awake state, while response latencies are more elongated [52]–[54]. Response to adjacent (non-principal) whiskers drops significantly in the sleep state compared to the awake state, suggesting less integration across neighboring barrels [10]. Therefore, we expect that stronger across-whisker integration in the awake state would be manifested by an increase in the across-barrel connectivity compared to the sleep state. In addition, the elevated firing rate of neurons recorded in the awake state is expected to enhance the statistical significance of the results and may provide a sharper estimate of networks with less overlap across whisker representations.

Studies of individual neuron responses to multi-whisker deflection suggest that single-whisker responses are superimposed, but not necessarily linearly [55]–[57]. It remains to be investigated, however, whether network responses to multi-whisker movements could be similarly represented as a superposition of the corresponding individual whisker-specific networks. Evidence in the literature suggest that supralinear summation of AMPA-mediated EPSPs and an increase in recurrent inhibition caused by incremental recruitment of inhibitory interneurons may have significant effect on the sensitivity and dynamic range of recurrent S1 circuits [58], [59]. It would be critical therefore to identify the neuron type in the inferred network graphs to ascertain that superposition of responses takes place. In addition, studying intra- and trans-laminar circuitry would be needed to assess how any disproportionate increase in inhibition with increase in excitation – for example, when multiple whiskers move simultaneously - is represented in the network graphs. We nonetheless expect that the net outcome of the network responses to multi-whisker movements should conform to a sparse network coding dynamics, consistent with numerous studies in other sensory areas. Such sparse code is important to maintain a time scale-dependent correlated activity that varies with the relative distance between neural elements [25].

It is noteworthy that the recurrent nature of cortical circuits, particularly those present in layer V, makes it especially difficult for pair-wise connectivity measures such as cross-correlograms to differentiate between direct and indirect coupling, such as in a neuronal chain, or when a common input is present. The DBN approach overcomes these limitations, as it integrates immediate evidence with prior information (long-term knowledge) and uses this process to explain away unlikely causes of firing [17]. In addition, DBN does not assume a specific model of the firing probability for the observed neurons.

Finally, it is important to bring our findings in perspective with two related hypotheses, namely cell-assemblies and synfire chains [60], [61]. Integration across neighboring columns as demonstrated by our analysis seems to support the cell assembly hypothesis, given the active participation of the observed neurons in whisker-specific networks. Participation of neurons in *transient* assemblies that are task/stimulus-dependent have been hypothesized to occur in multiple brain areas [32], [62], [63]. This implies that each postsynaptic neuron in the transient assembly *reads* patterns of firing of its pre-synaptic peers within temporal integration windows with possibly variable lengths. This is particularly interesting, in part because cell assemblies that reflect different degrees of synchrony between their elements have been hypothesized to correlate with presumed ‘top-down’ processing of sensory information [45], [64]. Herein, our findings suggest that similar mechanisms occur during bottom-up processing where sensory information is propagated to upstream cortical networks, and that coordination among the cells at millisecond timescale is a more wide spread phenomenon than previously thought.

## Materials and Methods

### Ethics Statement

All procedures involving animals were approved by the Michigan State University Institutional Animal Care and Use Committee (IACUC) (Animal Use Forms number 07/07-102-00 and 05/10-054-00).

### Barrel Cortex Recording

Five adult female Sprague Dawley rats weighing ∼300 g were used in this study (Rats R1–R5). Animals were anesthetized using a cocktail of ketamine and xylazine (75 and 5 mg/kg injected intrapertoneally, respectively). The left somatosensory cortex was exposed (4 x 4 mm craniotomy, 0–4 mm posterior and 4–8 mm lateral to bregma). A 32-channel microelectrode silicon array (NeuroNexus Technologies, Ann Arbor, MI, USA) with 4 shanks, 8 recording sites/shank, 400 µm shank separation and 100 µm electrode separation within shank was advanced into the barrel field in 100 µm steps. Acquired signals were amplified and band-pass filtered in the range 300–5000 Hz and sampled at 25 KHz. Stimulus-driven activity was recorded at depths of 1100–1500 µm corresponding to layer V of the barrel cortex. Subjects were perfused at the end of the experiments using 0.1 M phosphate-buffered saline and 4% paraformaldehyde. Coronal sections (50 *µ*m) were cut and sections were Nissl-stained. The laminar depth of the arrays was confirmed to be in layer V by examining either the length of the electrode tracks or electrolytic lesions created by passing 4 µA current for 5 sec (Figure S1).

Prior to vibrissae stimulation, whiskers were all trimmed to 6 mm length. For each rat, 3 whiskers were selected for mechanical stimulation that resulted in maximal modulation of the firing rate based on the observed neuronal response to manual deflection (Whiskers C2, C3 and D2 for R1; C2, D2 and D1 for R2; C1, D1 and D2 for R3; B2, B3 and B4 for R4; B1, B2 and B3 for R5). The selected whiskers were deflected one at a time by inserting each whisker into a capillary tube glued to a piezoelectric bimorph (Piezo Systems, Cambridge, MA, USA). Each whisker was horizontally deflected 900 times with a displacement of 80 µm for 100 ms (rise time and fall time were each set to 1 ms) at 1 Hz frequency [4], [65].

Spikes in multiple single unit activity were detected and sorted using NeuroQuest; a MATLAB toolbox for neural data processing and analysis [66]. Spikes presence was confirmed if the raw waveform surpassed a threshold set at 3 times the noise standard deviation. Due to the observed overlap in the recorded spikes in the data, a short spike length of 0.5 ms was used for spike sorting (0.25 ms pre threshold crossing and 0.25 ms post threshold crossing). Our analysis appeared not to be affected by the spike length (Figure S2). Spikes were aligned at their trough. Principal Component Analysis (PCA) was applied to the detected spikes, and the first 2 principal components were used as features for spike sorting. An average population size of 16±7.8 single units/rat was recorded (12 units for R1, 27 for R2, 21 for R3, 8 for R4 and 12 for R5). Spike trains were binned at Δ = 0.5 ms. Quality of the spike sorting was assessed using inter-spike interval histogram (ISI) to ensure that no spikes with inter-spike intervals of less than 1.5 ms were classified as belonging to the same unit. Neurons were indexed by channel number (1–32) and unit number (a, b, c, etc …).

**Single Unit Analysis**

For each neuron, Post-stimulus Time Histograms (PSTHs) were computed as the average firing across trials for each stimulated whisker with 0.5 ms bin size within a window of 100 ms post stimulus onset. The peak PSTH count for a given neuron for each whisker was then extracted. The mean first-spike latency *L_iw_* of each neuron *i* for a given whisker *w* was computed as[4], [29]

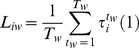
(1)


where *T_w_* is the total number of trials for whisker *w* and 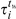
is a vector of the spike times of neuron *i* on trial *t_w_* relative to stimulus onset.

We quantified the amount of information present in the individual neuron and population response property, namely the first-spike latency, spike count and network graph representation, about the deflected whisker identity using mutual information [4], [31]. For a given neuron *i*, the mutual information between its response property *X_i_* and the stimulus *W* (in our case the whisker identity) was computed as
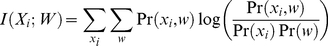
(2)


where *X_i_* corresponds to the time stamp of the first spike of neuron *i* within 100 ms of the stimulus onset in the case of first-spike latency, *X_i_* corresponds to the total number of spikes fired by neuron *i* during the same time interval in the case of spike count, and *X_i_* corresponds to the subnetwork of neuron *i* in the case of network representation as detailed later. The more distinct the response distribution of a given neuron to different whiskers is, the higher the mutual information, and thus, the higher the information it conveys about the identity of the deflected whisker. To normalize *I*(*X_i_*;*W*) between 0 and 1, we divided equation (2) by the joint entropy *H*(*X_i_*,*W*).

### Dynamic Bayesian Networks Analysis

Dynamic Bayesian Networks (DBNs) are graphical models used to fit spike train data. In these models, a directed acyclic graph (DAG) [18], denoted by *G*, and a set of conditional probabilities, denoted by *P*, represent the statistical dependence between the simultaneously observed spike trains (*r_1_, r_2_,…, r_n_*), and are used to represent the network *B* as *B = <G, P>*. Each graph *G* consists of a set of nodes *V* and edges *E*. Each node in *V*, denoted by *v_i_*(*t*), corresponds to the spike train of neuron *i* at time *t*, where *r_i_*(*t*)  = 1 represents a ‘spike’, and *r_i_*(*t*) = 0 represents ‘no spike’. Each directed edge in *E*, denoted by *v_i_*(*t*_1_)→ *v_j_*(*t*_2_), indicates that *r_j_*(*t*_2_) is conditionally dependent on *r_i_*(*t*_1_).

The state of each variable *r_i_*(*t*) in a DBN is determined only by its putative pre-synaptic cells' history, denoted **r** *_π_*_(*i)*_(1:*t-*1), and is independent of the state of any other cell. Thus, the probability 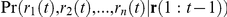 can be expressed in terms of the conditional probabilities Pr(*r_i_*(*t*)|**r** *_π_*_(*i)*_(1:*t-*1)) as
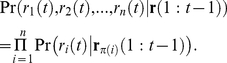
(3)


Learning DBN structure from the data can be achieved by searching for the structure G* that maximizes the posterior density of the network structure *G* for a given dataset *D*, denoted Pr(*G*|*D*), expressed using Bayes' rule as
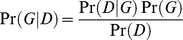
(4)


where Pr(*D*|*G*) is the likelihood of the data *D* given the structure *G*, Pr(*G*) is the structure prior, and Pr(*D*) is the probability of the observed data. Assuming a uniform distribution for Pr(*G*) (i.e. no prior information about the structures) and given that Pr(*D*) is independent of the choice of *G*, G* can be found by maximizing Pr(*D*|*G*). A closed form for Pr(*D*|*G*) can be obtained under Dirichlet distributed priors [67]. A search is then carried out through the space of all possible structures to find the model with maximum likelihood.

To infer whisker-specific networks, we used the Bayesian Network Inference with Java Objects (BANJO) toolbox [19] with simulated annealing search algorithm [68]. For each deflected whisker, spike trains within 100 ms of each stimulus onset were considered as one trial. A total of 100 datasets, 18 sec each, for each whisker were extracted from the recorded 900 trials/whisker, where each dataset was formed by concatenating 180 trials that were randomly chosen with a uniform distribution from the 900 trials. This results in an overlap between any given pair of datasets that follows a binomial distribution with a mean overlap of 20% and a standard deviation of 4% (Figure S3). The spike trains of each dataset were analyzed using DBN with Markov lags in the range [1], [10] bins ([0.5, 5] ms). A Markov lag range of [1], [5] bins ([0.5, 2.5] ms) was found to be the best range for all datasets based on calculations of an *influence score* that measures the degree of influence each pre-synaptic cell has on post-synaptic cells [17], [69]. In case a connection was inferred at more than one Markov lag, only the largest lag was considered. The maximum number of pre-synaptic cells for each cell was set to 10.

### Network Similarity and Network Information

To quantify the similarity between the inferred networks, we first represented each inferred network as an *n* × *n* binary adjacency matrix *A*, where *n* is the total number of neurons in the network. Each element *A*(*i*, *j*) takes the value ‘1’ if there is a connection from neuron *i* to neuron *j* and ‘0’ if there is no connection between the corresponding neurons. For a given population of *n* neurons, *K* deflected whiskers and *M* datasets per whisker, all the adjacency matrices of the inferred networks were vectorized and stacked together into one *KM* × *n* ^2^ matrix. Principal component analysis (PCA) was then applied to this matrix to extract significant features from the inferred networks by projecting the adjacency matrices into a *p*-dimension network space, where *p*≤*n* ^2^, that accounts for most of the variance in the networks [35]. The similarity *R*(*A_l_*, *A_m_*) between a pair of adjacency matrices *A_l_* and *A_m_* was defined as
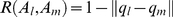
(5)


where *q_l_* and *q_m_* are the projections of *A_l_* and *A_m_* in the *p*-dimension network space, respectively, and ||.|| is the Euclidean distance (*l_p_*-norm) between the two projections. The network space was normalized such that the maximum possible distance between any pair of projections is 1. The number of principal components used *p* was set to 2. The average across-whiskers similarity 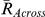 and the average within-whisker similarity 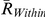 for a given population were therefore defined as
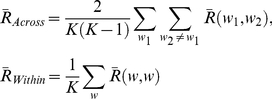
(6)


where the average similarity between the networks inferred for a given pair of whiskers *w*_1_ and *w*_2_, 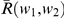, and within a given whisker *w*, 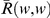, were defined as
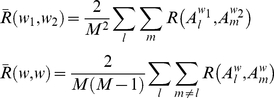
(7)


Similar to the expression given in equation (2), the mutual information between the network projection *Q* and the stimulus *W* was computed as
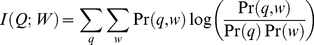
(8)


where the probabilities used in equation (8) were computed from the 2-dimensional network space after discretizing it into 10×10 bins. For the mutual information measure of individual neurons' subnetworks, *Q* corresponds to the projection of the subnetworks of each neuron onto the network space. The normalized *I*(*Q*;*W*) was computed by dividing equation (8) by the joint entropy *H*(*Q*,*W*). It is noteworthy that the estimated probabilities, and so the computed mutual information, vary with the discretization bin size. Our results, however, did not seem to be affected by the choice of the bin size (Figure S4).

In order to estimate any bias in 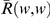 and *I*(*Q*;*W*) that results from the overlap in the datasets of each whisker, surrogate datasets were created from the original data such that each surrogate contains trials from *all* whiskers that has the same degree of overlap as the original datasets (Figure S3). Thus, within a surrogate, any excess similarity between the networks inferred for the datasets extracted from it would only result from the overlap between these datasets and not from the stimulus. To form the shuffled datasets, the 900 trials recorded for each whisker were first split into 3 different groups, 300 trials each. One group was then selected from each whisker and concatenated with 1 group from each of the other 2 whiskers forming a 900 trial shuffled surrogate (For example: group 1 of whisker 1, group 1 of whisker 2 and group 1 of whisker 3 form one shuffled surrogate; group 2 of whisker 1, group 2 of whisker 2 and group 2 of whisker 3 form another shuffled surrogate, … etc). For each subject, 3 shuffled surrogates, 900 trials each, were formed. A total of 100 datasets, 18 sec each, were then extracted from each shuffled surrogate by concatenating 180 trials that were randomly sampled from the 900 trials following a uniform distribution. Therefore, these datasets were not whisker-specific and thus any similarity between the networks inferred within the same shuffled surrogate that is larger than that across surrogates would only result from the overlap between the datasets extracted from the same surrogate. Similarly, any non-zero mutual information between the networks inferred for the shuffled surrogates and the identity of these surrogates would also result from the overlap between the datasets extracted from the same surrogate. We used the difference between the within-surrogate similarity and the across-surrogates similarity as an estimate of the bias in the within-whisker similarity of the original data that results from the overlap. We also used the mutual information computed for the shuffled datasets as an estimate of the bias resulting from the overlap.

### Decoding Whisker Identity

We used a leave-one-out cross-validation approach to test the ability to decode the identity of the deflected whisker using the inferred networks [70]. The network obtained for each dataset of a given whisker was compared to the other networks inferred for the same whisker (*M* - 1 networks) and the other whiskers (*M*(*K*-1) networks). The identity of the deflected whisker w* for a given test dataset was computed as
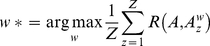
(9)


where *Z* = *M* when w ≠ w* or *Z* = *M*−1 when *w* = w* (to exclude the test dataset network from the similarity comparison). Therefore, w* is the whisker whose inferred networks (templates) result in maximum similarity with the test data fit *A*.

Decoding based on the response latency of individual neurons for a given test dataset was obtained as follows: we first computed the mean first-spike latency for each neuron from all datasets of all whiskers excluding the test dataset (training datasets). The mean first-spike latency for each neuron in the test dataset was computed and compared to that obtained from the training datasets. Whiskers whose training datasets for each cell had *the least* absolute latency difference were identified as the ones being stimulated. Single-cell decoding accuracy was then computed as the percentage of test datasets for which the decoded whisker identity matched the actual whisker. The overall decoding accuracy was computed by averaging across cells. For comparison, we also computed the decoding accuracy using a majority-voting rule in which whisker identity was determined as the one with the majority of cells having the least absolute latency difference between training and test datasets.

### Cross-correlogram Analysis

For the sake of comparison, we computed the standard cross-correlogram approach to assess potential causal influence between pairs of neurons with bin size of 0.5 ms and range [−5, 5] ms [36]. Ten jittered versions of each of the datasets analyzed using DBN were formed in which each spike was randomly displaced over a uniform interval in the range [−10, 10] ms around the original spike time. A peak or trough (indicating excitation or inhibition, respectively) in the cross-correlogram for a given neuron pair in the original dataset was determined to represent a connection if it crossed a confidence level computed from the jittered datasets. The upper and lower limits of the confidence level were computed from the maximum and minimum counts of the jittered cross-correlograms, respectively, with an acceptance level of 0.99 [32].

### Predicting Single Neuron Firing from Putative Pre-synaptic Peers

The firing probability of each neuron was estimated using the firing history (5 bins or 2.5 ms) of pre-synaptic cells determined from the networks inferred for each whisker and the stimulus. The conditional firing probability of a given neuron *i* at time *t* was estimated as
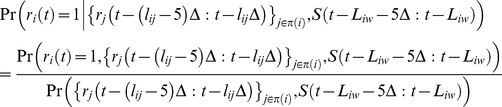
(10)


where *π*(*i*) is the set of pre-synaptic neurons inferred for whisker *w*, *l_ij_* is the Markov lag at which a connection from neuron *j* to neuron *i* was inferred, *S*(*t*) is a binary vector with a nonzero entry of ‘1’ only at the stimulus onset and 0 otherwise, and *L_iw_* is the mean first-spike latency computed in equation (1). Using ten-fold cross-validation, 10 training datasets (72 sec duration each) were extracted from each 90 sec whisker dataset by sliding a 72 sec window with 5 sec steps. The joint probabilities on the right hand side of equation (10) were computed from each training dataset using kernel density estimation with a normal function kernel and a bandwidth of 0.001 [71]. The probabilities estimated from each training dataset were used to predict the firing of each neuron in the remaining 18 sec test dataset. When conditioned on the stimulus only, equation (10) can be re-written as
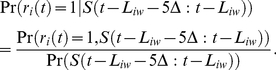
(11)


The probability estimates were then smoothed by applying a moving average filter with a window of 15 ms. Predicted spike trains were computed for test datasets (10 datasets/whisker, 18 sec each) using the smoothed estimates for threshold values in the range [0, 1] with a step of 0.001. At each time point, the original spike trains were used in the prediction. True and false positive rates were computed from the predicted spike trains for each threshold. Receiver Operating Characteristic (ROC) curves were constructed from the true and false positive rates for each cell for a given whisker. The area under the ROC curve was used as a measure of the predictive power as 2× (Area under ROC – 0.5) [37].

We compared the predictive power using the DBN method to that obtained using Generalized Linear Models (GLMs) [38]. GLM expresses the firing probability of a neuron *i* as
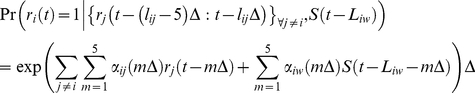
(12)


where *α_ij_* models the coupling filters between neurons *i* and *j* and *α_iw_* models the stimulus filter. The history interval was set to 5 bins (2.5 ms) similar to the DBN analysis. The coupling and stimulus filters were estimated using the same training datasets for the DBNs using iterative reweighted least squares (IRLS). These filters were then used to compute the firing probability of each neuron at each time point of the test datasets. Spike train predictions were computed by comparing the estimated firing probability to threshold values in the range [0, 1]. ROC curves were constructed and the predictive power was also computed for each neuron for a given whisker.

## Supporting Information

Figure S1 **Nissl stained coronal section (50 µm) in rat R5.** This rat was chronically implanted over 35 days. Dashed curve indicates the original shape of the section that was damaged during the removal of the implant. Black arrowhead points to an electrolytic lesion mark of the deepest recording site on one of the shanks of the multi-electrode array. The depth of the lesion mark (∼1250 µm) is consistent with the depth recorded using the micromanipulator during the surgery and corresponds to layer Vb of the barrel cortex (1.1 mm posterior and 5.2 mm lateral to bregma).
(TIF)

Figure S2 **Variability in spike length and bias.** Using a spike length of 1 ms during spike sorting and a spike train bin width of 1 ms for rat R5 did not bias the results. (A) Similarity between networks inferred for the same whisker (left) and networks inferred for different whiskers (right) for the same population (mean ± SD). * *P*<0.001, two-sample *t*-test. Similar to Figure 3B, more similarity is observed between within-whisker networks compared to across-whisker networks. (B) Histogram of the difference between the mean first-spike latency of the post-synaptic cells and the pre-synaptic cells for each inferred connection. Only unidirectional connections were counted in the histogram. Red bars indicate the fraction of connections consistent with the difference between the latencies while blue bars indicate connections that are not. The majority of inferred connections (85.8%) were from neurons with smaller absolute latencies to neurons with larger absolute latencies similar to Figure 5A. (C) The ratio between the number of outgoing connections and incoming connections for each neuron (source index) as a function of its mean first-spike latency for each whisker. Z-scores of the mean first-spike latency and the source index are reported on the X-axis and the Y-axis, respectively. Gray curve indicates decaying exponential fit. The source index decays exponentially with the mean first-spike latency (Time constant  = −0.7, *r* ^2^ = 0.04, *n* = 36) similar to Figure 5B.
(TIF)

Figure S3 **Overlap in the original and shuffled datasets.** (A) Distribution of the amount of overlap between any pair of datasets for (Right) the original data and (Left) the shuffled data. Blue curve indicates a binomial distribution fit with parameters *p* = 0.2 and *n* = 180. Both figures indicate that both the original and the shuffled data have the same degree of overlap, where any given pair of datasets would have an overlap of 20±4%. (B) Network feature space of the shuffled datasets extracted from rat R2 data. Each dot corresponds to the projection of one network onto a 2-dimensional principal components (PC1 and PC2) feature space. (C) Similarity between networks inferred for the same shuffled dataset and between networks inferred for different shuffled datasets averaged across subjects (mean ± SD). Similarity for a given pair of networks was quantified as 1 – the distance between the projections of the two networks in the principal component feature space.
(TIF)

Figure S4 **Network information in the original data is consistently higher than that in the shuffled data, independent of the bin size.** (A) Normalized network information in the original and the shuffled data as a function of the bin size used to estimate the mutual information averaged across the 5 subjects (mean ± SD). (B) Normalized information in the network of individual neurons computed from the original and the shuffled data as a function of the bin size used to estimate the mutual information, averaged across 80 neurons (mean ± SD).
(TIF)
