# Supplementary figures and images for: Millisecond-Timescale Local Network Coding in the Rat Primary Somatosensory Cortex
Source: PLoS One. 2011 Jun 29;6(6):e21649. doi: 10.1371/journal.pone.0021649 (PMC3126857; doi:10.1371/journal.pone.0021649)

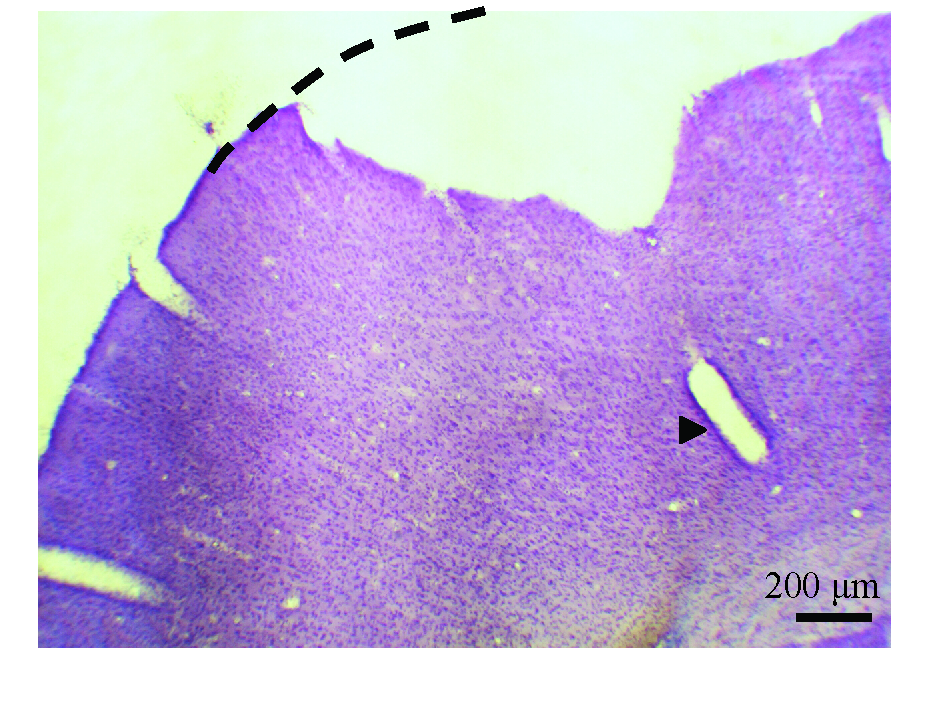

Supplement: Figure S1 — Nissl stained coronal section (50 µm) in rat R5. This rat was chronically implanted over 35 days. Dashed curve indicates the original shape of the section that was damaged during the removal of the implant. Black arrowhead points to an electrolytic lesion mark of the deepest recording site on one of the shanks of the multi-electrode array. The depth of the lesion mark (∼1250 µm) is consistent with the depth recorded using the micromanipulator during the surgery and corresponds to layer Vb of the barrel cortex (1.1 mm posterior and 5.2 mm lateral to bregma). (TIF) [file pone.0021649.s001.tif]

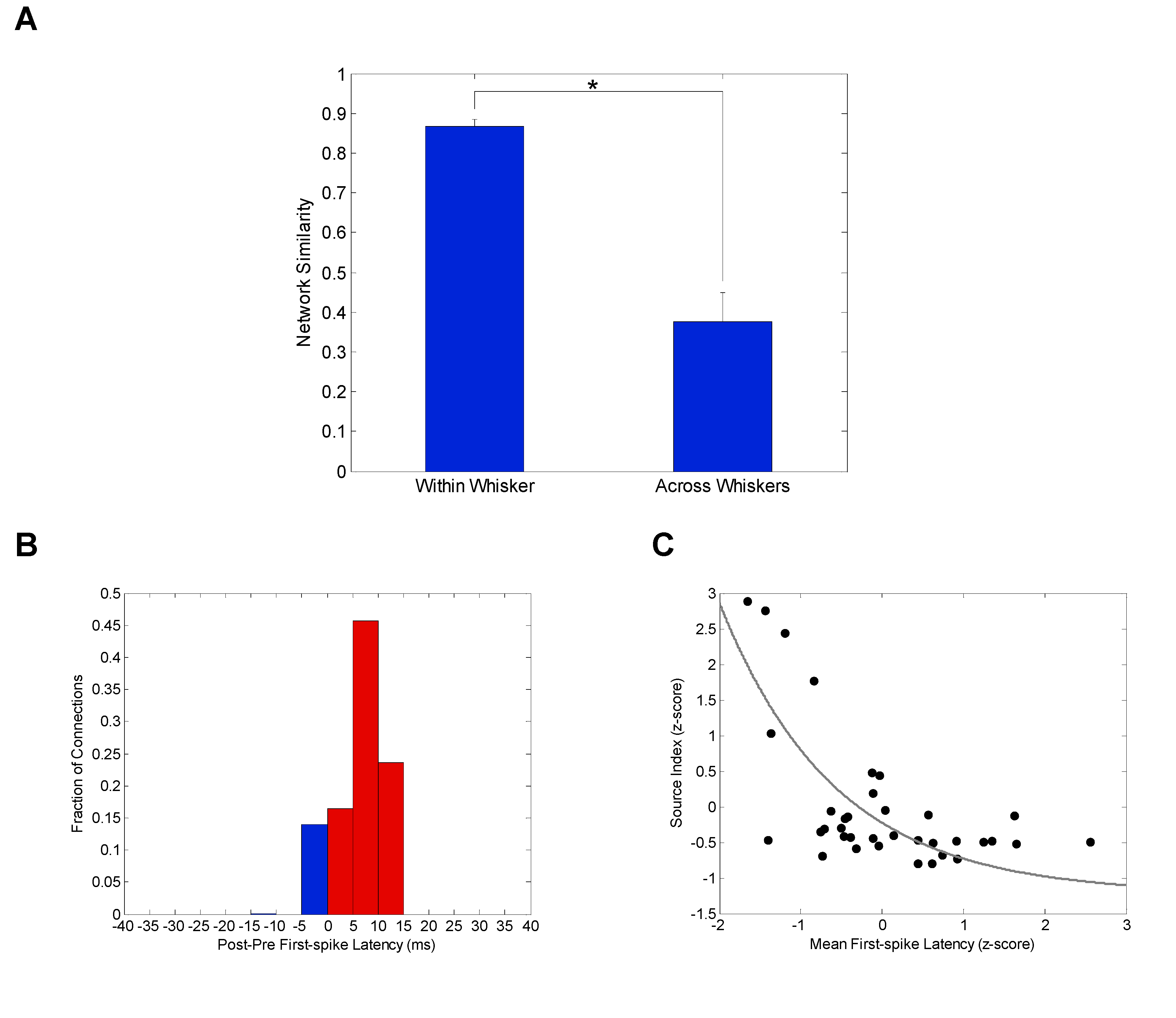

Supplement: Figure S2 — Variability in spike length and bias. Using a spike length of 1 ms during spike sorting and a spike train bin width of 1 ms for rat R5 did not bias the results. (A) Similarity between networks inferred for the same whisker (left) and networks inferred for different whiskers (right) for the same population (mean ± SD). * P<0.001, two-sample t-test. Similar to Figure 3B, more similarity is observed between within-whisker networks compared to across-whisker networks. (B) Histogram of the difference between the mean first-spike latency of the post-synaptic cells and the pre-synaptic cells for each inferred connection. Only unidirectional connections were counted in the histogram. Red bars indicate the fraction of connections consistent with the difference between the latencies while blue bars indicate connections that are not. The majority of inferred connections (85.8%) were from neurons with smaller absolute latencies to neurons with larger absolute latencies similar to Figure 5A. (C) The ratio between the number of outgoing connections and incoming connections for each neuron (source index) as a function of its mean first-spike latency for each whisker. Z-scores of the mean first-spike latency and the source index are reported on the X-axis and the Y-axis, respectively. Gray curve indicates decaying exponential fit. The source index decays exponentially with the mean first-spike latency (Time constant = −0.7, r 2 = 0.04, n = 36) similar to Figure 5B. (TIF) [file pone.0021649.s002.tif]

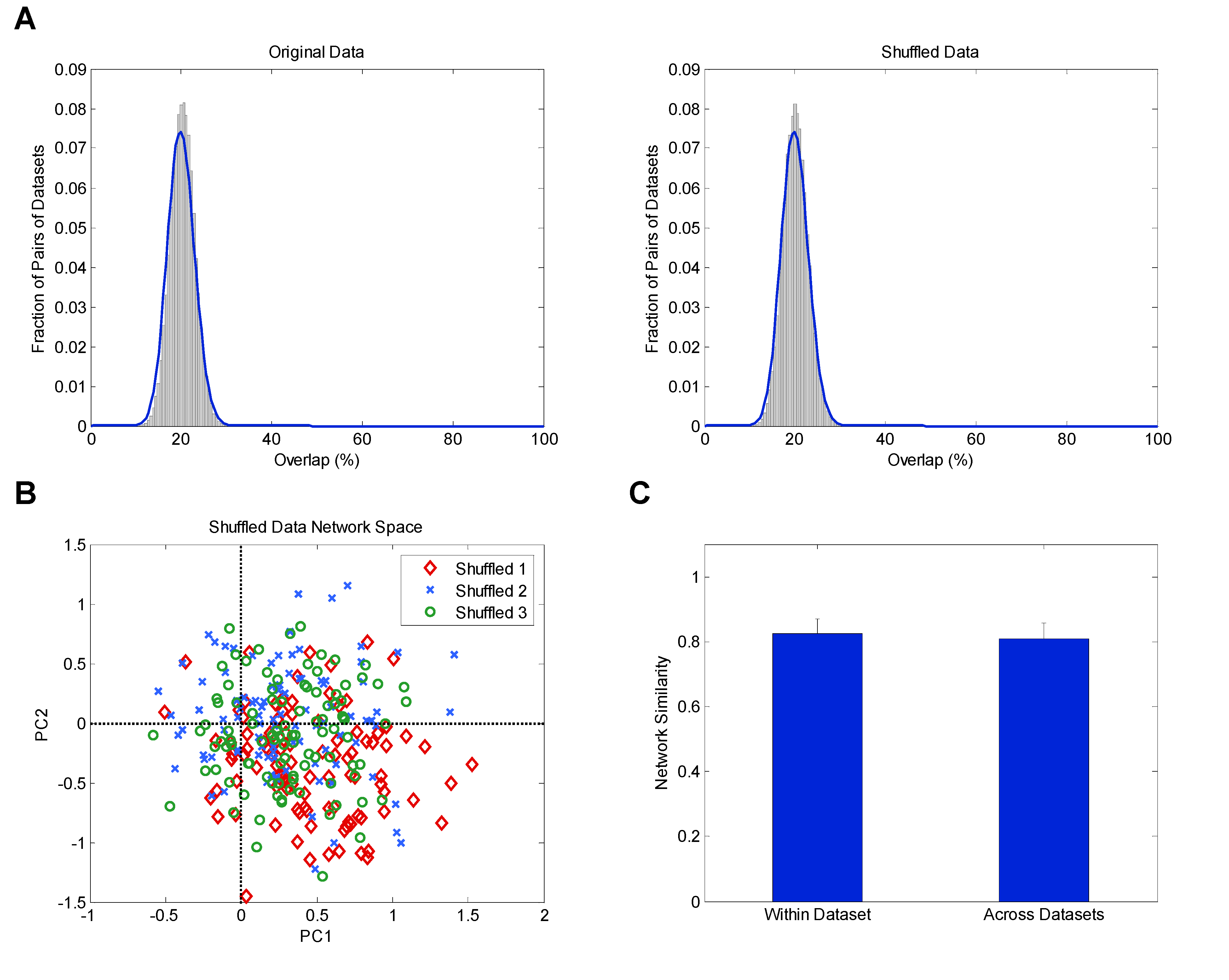

Supplement: Figure S3 — Overlap in the original and shuffled datasets. (A) Distribution of the amount of overlap between any pair of datasets for (Right) the original data and (Left) the shuffled data. Blue curve indicates a binomial distribution fit with parameters p = 0.2 and n = 180. Both figures indicate that both the original and the shuffled data have the same degree of overlap, where any given pair of datasets would have an overlap of 20±4%. (B) Network feature space of the shuffled datasets extracted from rat R2 data. Each dot corresponds to the projection of one network onto a 2-dimensional principal components (PC1 and PC2) feature space. (C) Similarity between networks inferred for the same shuffled dataset and between networks inferred for different shuffled datasets averaged across subjects (mean ± SD). Similarity for a given pair of networks was quantified as 1 – the distance between the projections of the two networks in the principal component feature space. (TIF) [file pone.0021649.s003.tif]

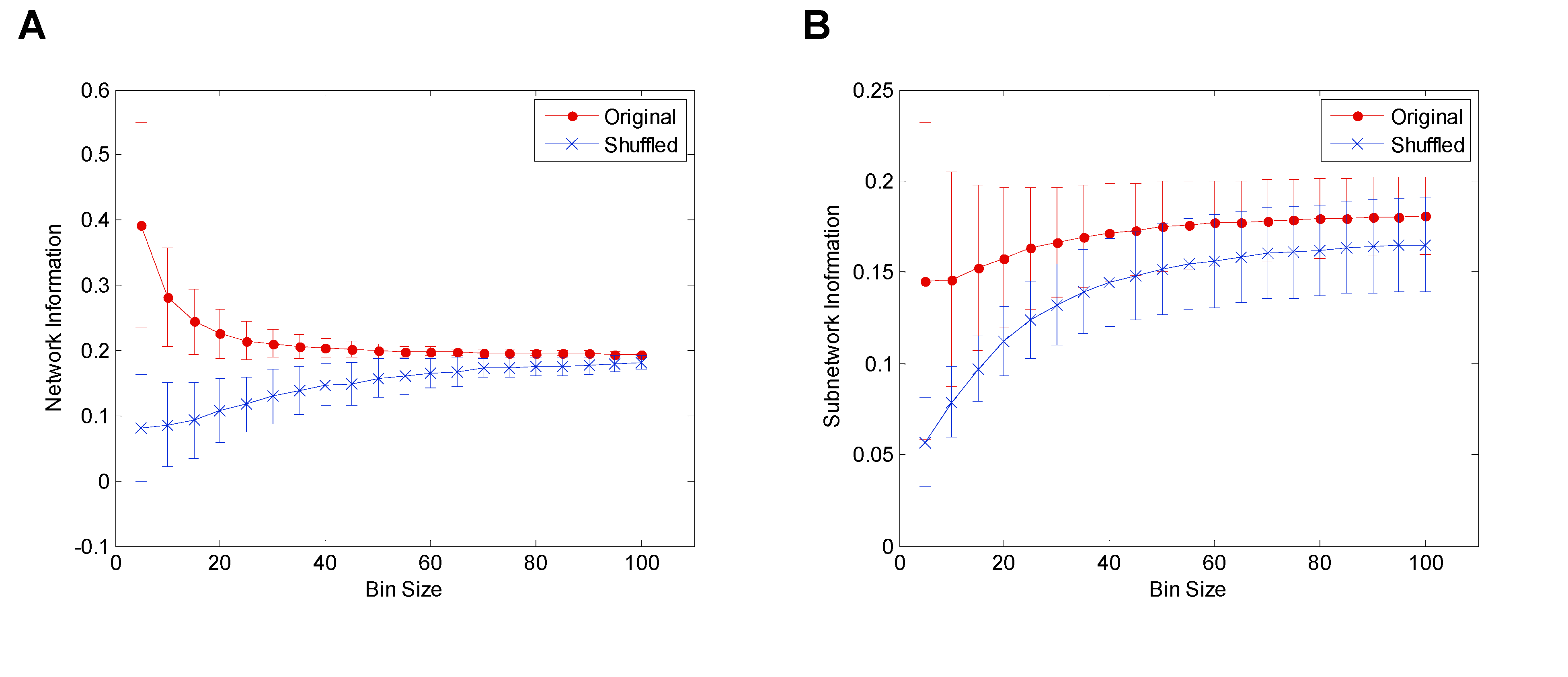

Supplement: Figure S4 — Network information in the original data is consistently higher than that in the shuffled data, independent of the bin size. (A) Normalized network information in the original and the shuffled data as a function of the bin size used to estimate the mutual information averaged across the 5 subjects (mean ± SD). (B) Normalized information in the network of individual neurons computed from the original and the shuffled data as a function of the bin size used to estimate the mutual information, averaged across 80 neurons (mean ± SD). (TIF) [file pone.0021649.s004.tif]
